# Analysis of Big Data in Gait Biomechanics: Current Trends and Future Directions

**DOI:** 10.1007/s40846-017-0297-2

**Published:** 2017-07-17

**Authors:** Angkoon Phinyomark, Giovanni Petri, Esther Ibáñez-Marcelo, Sean T. Osis, Reed Ferber

**Affiliations:** 1 ISI Foundation, Via Chisola 5, Turin, 10126 Italy; 2 Faculty of Kinesiology, University of Calgary, 2500 University Dr NW, Calgary, AB T2N 1N4 Canada; 3 Faculty of Nursing, University of Calgary, 2500 University Dr NW, Calgary, AB T2N 1N4 Canada; 4 Running Injury Clinic, University of Calgary, 2500 University Dr NW, Calgary, AB T2N 1N4 Canada

**Keywords:** Data science, Biomechanics, Gait, Kinematics, Principal component analysis, Support vector machine, Topological data analysis

## Abstract

The increasing amount of data in biomechanics research has greatly increased the importance of developing advanced multivariate analysis and machine learning techniques, which are better able to handle “big data”. Consequently, advances in data science methods will expand the knowledge for testing new hypotheses about biomechanical risk factors associated with walking and running gait-related musculoskeletal injury. This paper begins with a brief introduction to an automated three-dimensional (3D) biomechanical gait data collection system: 3D GAIT, followed by how the studies in the field of gait biomechanics fit the quantities in the 5 V’s definition of big data: volume, velocity, variety, veracity, and value. Next, we provide a review of recent research and development in multivariate and machine learning methods-based gait analysis that can be applied to big data analytics. These modern biomechanical gait analysis methods include several main modules such as initial input features, dimensionality reduction (feature selection and extraction), and learning algorithms (classification and clustering). Finally, a promising big data exploration tool called “topological data analysis” and directions for future research are outlined and discussed.

## Introduction

Biomechanical gait analysis is commonly used to analyse sport performance and evaluate pathologic gait. Significant advances in motion capture equipment, research methodologies, and data analysis techniques have enabled a plethora of studies that have advanced our understanding of gait biomechanics. Despite these advances, however, much of the biomechanical research over the past 20 years has investigated the influence of potential injury risk factors in isolation [1]. More likely, multiple biomechanical and clinical variables interact with one another and operate as combined risk factors to the point that traditional biomechanical analysis methods (e.g., analysis of several discrete variables, such as peak angles, together with a statistical hypothesis test, such as *t* test or the analysis of variance (ANOVA) [2, 3]) cannot capture the complexity of these relationships. In response to these shortcomings, advanced multivariate analysis and machine learning methods such as principal component analysis (PCA) and support vector machine (SVM) have been used to identify these complex associations [2, 3]. However, to build accurate classification models, an adequate number of samples is needed, which grows exponentially with the number of features used in the analysis [4]. Therefore, to directly meet this need the University of Calgary group (Ferber, Osis) have developed the infrastructure and established a worldwide and growing network of clinical and research partners all linked through an automated three-dimensional (3D) biomechanical gait data collection system: 3D GAIT. Considering that traditional data analytics may not be able to handle these large volumes of data, appropriate “big data” analysis methods must also be developed [5, 6].

This paper begins with an introduction to the 3D GAIT system, followed by an overview of a big data problem in gait biomechanics using the 5 V’s: volume, velocity, variety, veracity, and value [7]. Next, a comprehensive overview of existing methods on the role of big data analytics is presented. We discuss the main components of modern biomechanical gait analysis involving: initial input features, dimensionality reduction using feature selection and feature extraction, and learning algorithms via classification and clustering. Finally, a promising big data exploration tool called “topological data analysis” is discussed along with future research directions.

## Data Collection System

**Fig. 1:**
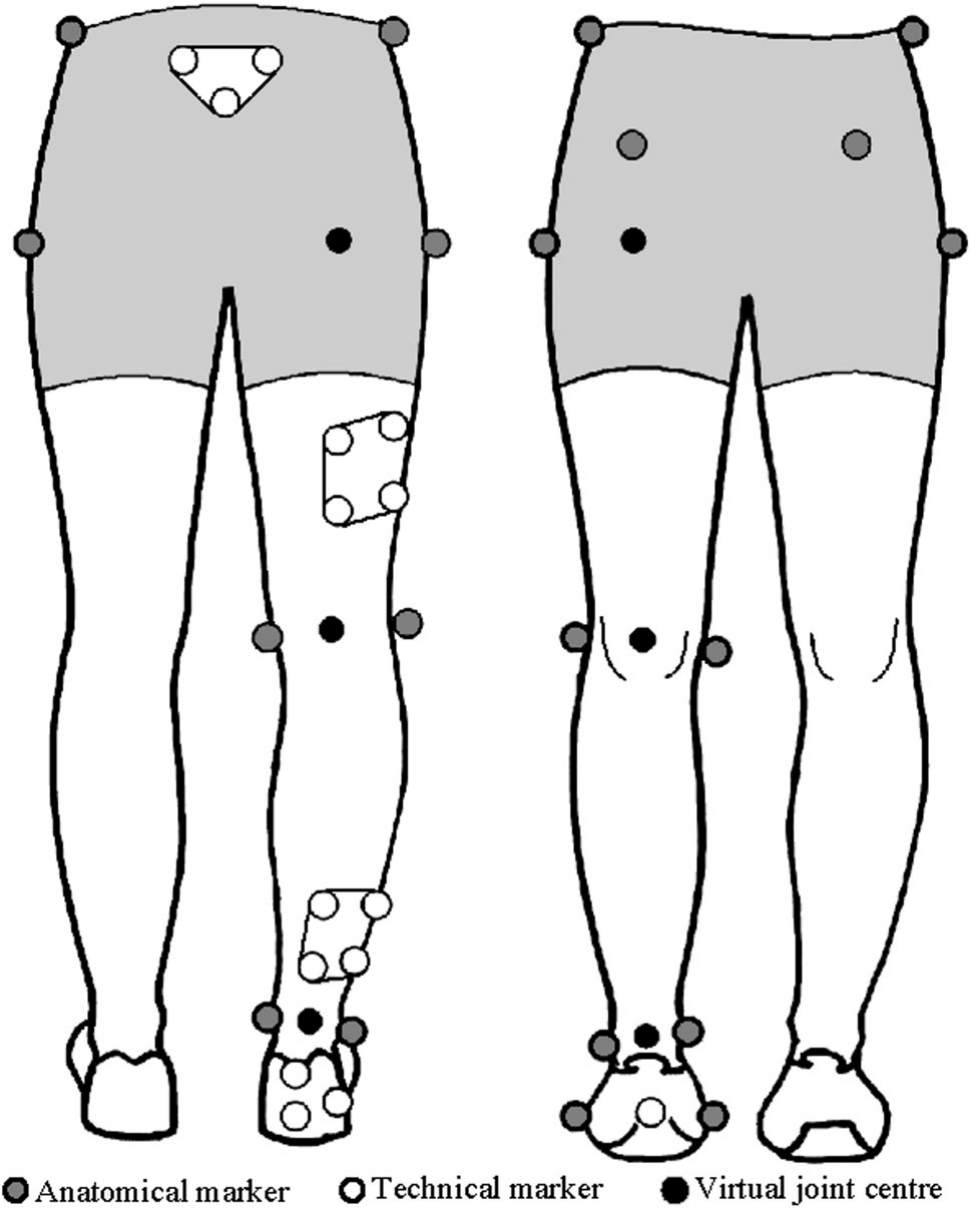
Marker placement for standard biomechanical gait analysis

The 3D GAIT system is a deployed turnkey motion capture platform specifically designed for gait analysis using a treadmill. The overall system design is a nexus of three main principles: ease-of-use/automation, biomechanics best practices, and data science best practices. Consequently, the system uses off-the-shelf passive motion capture technology, consisting of between three and six infrared cameras (Vicon Motion Systems, Oxford) along with spherical retroreflective markers that are pre-configured for ease of placement on the subject. Rigid clusters of markers are strapped to the subject’s thighs, shanks and pelvis, and markers are taped to the shoes to define foot movement (Fig. 1). Additional markers are also placed on the specific anatomical landmarks and are used to define the location of joint centers. During a treadmill session, the cameras operate at 200 Hz for 30 s, collecting ~150,000 data points representing the 3D coordinates of each marker. These marker data are then transformed using rigid-body kinematics [8, 9] into joint angles, which are 3D representations of body movements, between segments, over time.

Joint angles from treadmill gait represent a set of non-independent, time-series waveforms, and there are several types of analyses that can be undertaken. In terms of biomechanics best practices, it is often considered appropriate to determine a “characteristic” pattern of motion that is representative of the movements for a given subject. Therefore, the 3D GAIT system derives a “characteristic” pattern from a spatio-temporal normalized set of gait cycles, which are segmented using a machine learning approach to account for inter-subject variability in technique [10, 11]. These normalized gait cycles can then be analyzed by: (1) collapsing into a single representative time-series data set by various averaging techniques (i.e., median, weighted nearest-neighbor interpolant), and/or (2) extracting discrete features from each cycle separately, and merging into a representative feature set for a given subject (i.e., median peak angle, mean angular excursion). In the latter case, 3D GAIT software generates a feature vector of 74 variables for both left and right sides, representing a substantial number of dimensions in the final data set. These feature vectors and time-series curves are then presented to the user to inform assessment, rehabilitation and training tasks.

After processing, and according to best practices in data science, the final data set is anonymized and packaged for transport. Marker data from the motion capture system, along with biomechanical feature vectors and demographic information (i.e., height, mass, age, etc.) are securely transmitted via end-to-end encryption to the central server for further processing and storage in a research database. These aggregate data, along with critical subject characteristics, allow the potential to statistically model lower limb injury and disease outside of the laboratory setting. Post-hoc analysis also provides the opportunity to develop updated biomechanical models and techniques, which can then inform future modifications to the deployed software.

Despite advances in 3D motion analysis, there are some limitation inherent within any 3D gait analysis methodology. For example, variability in kinematic variables may be attributed to measurement error, skin marker movement, marker re-application errors, and inherent physiological variability during human locomotion. While the first three factors are independent of the patient population itself, it is possible that physiological variability may be different in a clinical population. Moreover, set-up and operation of 3D gait systems requires calibration by an trained expert, and the time required to operate these systems limits its practicality, especially in a clinical setting.

## Big Data Characteristics

There is no universal definition of the term “big data” and a rough definition would be datasets whose size or structure are beyond the ability of traditional data collection and analysis tools to process, within a reasonable time [5, 6]. This definition also implies that many traditional analytics may not be able to be applied directly to big data. To sufficiently describe big data, and to incorporate other facets of its nature, the definition of big data has expanded beyond limitations of data size, to include several key attributes such as variety, velocity, veracity and value, in order to propose a new complete definition of the term “big data” (e.g. 3 V’s [12] and 5 V’s [7]). Data gathered for biomechanics research exhibits many of these qualities and can be considered to be “big data”, in the light of the 5 V’s definition, as follows.

### Volume (Quantity of Data)

Volume refers to vast amounts of data. While traditional biomechanical analysis generally involves only a few variables and low subject numbers, recent advancements in data collection technology generate more data for each subject, and modern biomechanical research is continually involving big volume datasets. Specifically, the number of variables per subject has increased to ~50–150 discrete variables [2, 3], several hundred to thousand variables for joint angle time-series data [13, 14], and several thousand to hundred thousand variables for marker coordinate time-series data [15, 16]. Although most of these studies continue to involve only a small cohort of subjects (e.g., 10–30 subjects [17, 18]) in the analysis, it is generally recommended to expand and further test proposed data science techniques and/or models using datasets with greater subject numbers to determine whether results are similar in different populations. The aforementioned research database can provide the necessary large cohort of subjects for such hypothesis-driven research (e.g., 483 subjects [2]).

### Variety (Different Data Categories)

Variety applies to data that exists in multiple types and/or captured from different sources. For example, recent biomechanical research involves data from 3D motion capture as well as clinical data such as self-reported pain/function and lab exams [19, 20]. These data would include continuous, discrete, and categorical data and thus sophisticated statistical methods need to be employed.

### Velocity (Fast Generation of New Data)

Velocity refers to the pace at which new data is created and collected. Walking and running gait related-injuries are often chronic in nature and rehabilitation often takes weeks-to-months. In order to monitor the progress of a rehabilitation program, gait data are generally collected at baseline, and some data are collected once a week over several weeks of the program [19, 20]. On average, 25 new patients are added each week to the UCalgary research database, and 12–15 new clinic partners are added each year.

### Veracity (Quality of Data)

Veracity refers to noisy, erroneous, or incomplete data and in biomechanics research this term can easily apply as data are often captured through different sensors and systems, as well involving measurement errors associated with kinematic data. Sources of error can result from many factors such as soft tissue artifact, electrical interference, and improper digitization and placement of retro-reflective markers. Although in general, there is a large divide between clinical research and clinical practice. Data from standard 3D motion capture systems are generally of high quality. Despite this, there is the possibility of incomplete clinical data, due to missing self-reports and lab exams. Fortunately, big data analytics can handle incomplete data sets when necessary using data science techniques such as *k*-nearest neighbors to impute the missing values [21].

### **Value** (In the Big Data)

Although the potential value associated with these complex and large data sets is very high, the real value of big data analytics in gait biomechanics still remains to be proven, and more sophisticated analytics, which incorporate a priori knowledge, are necessary. For instance, suitable techniques for extracting useful information can lead to high value outcomes even in situations with low data veracity. Further, multivariate analysis and machine learning methods could potentially be utilized as an automated system for detecting gait changes related to injury [3, 22]. However, more research is necessary to advance these ideas. It is also important to note that although not all the studies in the field of gait biomechanics fit all 5 V’s of big data, data lacking one of the attributes can still be analysed using complex data science methods.

## Initial Input Features

Most investigations of walking and running gait biomechanics involve kinematic data and have focused on determining gait waveform events such as joint angles at touchdown, toe-off, mid-stance, and mid-swing [23]. Descriptive statistics such as peak angles, excursion, and range of motion are also commonly extracted from the gait waveform [23]. However, these traditional approaches call for a priori selection of features, which relies on sufficient background knowledge and/or subjective opinion. Consequently, a large proportion of the kinematic data are discarded, whereas they may contain meaningful information related to the between-group differences.

**Fig. 2:**
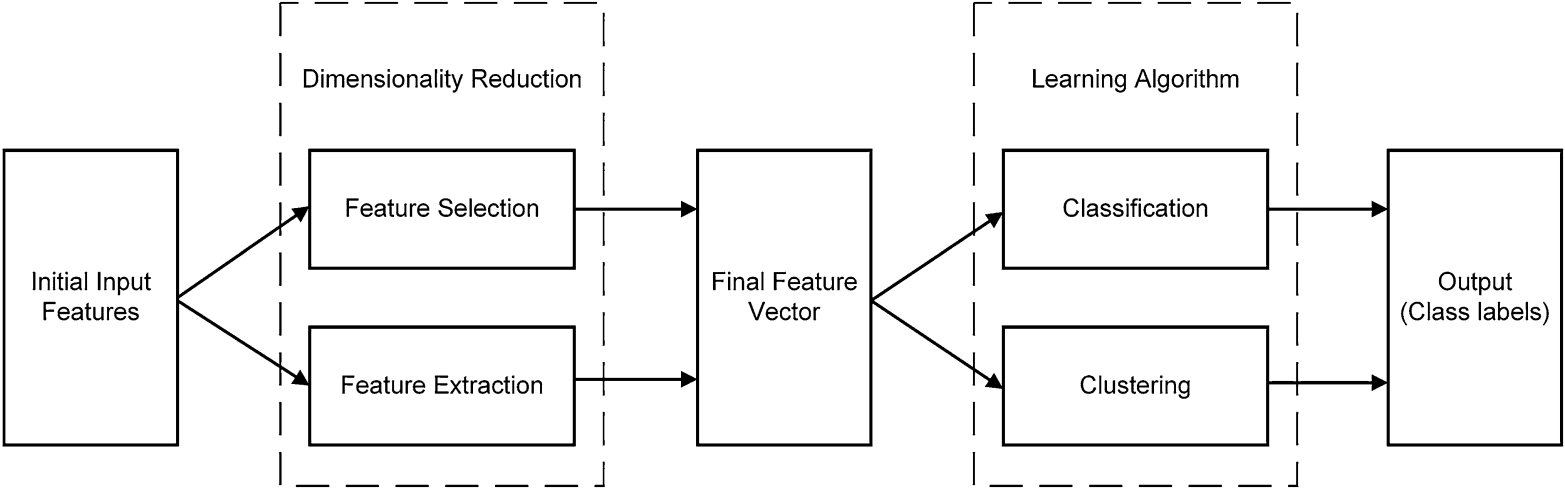
The main components of modern biomechanical gait analysis

In contrast, modern data science methods involve the following main components of initial input features, dimensionality reduction using feature selection and feature extraction, and learning algorithms via classification and clustering (Fig. 2). A summary of some previous contributions involving their clinical application has been presented in Table 1. By expanding initial input features, and analysing the entire gait waveform, new insights can be derived from the data to help improve clinical practice. For example, Phinyomark et al. [13] examined differences in gait kinematics between healthy runners as compared to runners with iliotibial band syndrome (ITBS), the second most common running-related injury and the most common cause of lateral knee pain, using the entire gait waveform together with a feature selection approach. They found that female ITBS runners exhibited greater hip external rotation angles during 56–58% of the running gait cycle while male ITBS runners exhibited significantly greater ankle internal rotation angles during 70–72% of the running gait cycle as compared with their respective healthy controls. The results suggest that clinicians should focus on strengthening proximal muscles for female runners and distal muscles for male runners to prevent ITBS injury, and these meaningful joint angles are obtained by examining the entire set of input data. Hence, either a set of representative variables [2, 3, 24] or the entire gait waveforms [13, 14] across joints and planes of motion should be employed as the initial set of input features.

**Table 1:** Summary of biomechanical gait analysis studies using data science methods and their research question of interest

Reference	Number of subjects	Initial input features	Dimensionality reduction	Leaning algorithms	Research question of interest
[13]	96	900 features (9 running kinematic waveforms)	MR (the best 3 angles/waveform)	–	Differences between male and female runners experiencing ITBS at the time of testing and healthy gender- and age-matched runners
[2]	483	72 features (running kinematic variables)	MR (the best 8–62 PCs)	SVM (78.4–100%)	Gender- and age-related differences in healthy runners
[27]	34	31 features (running kinematic variables)	SFS (the best 6 features)	SVM (100%)	Age-differences in healthy runners
[40]	92	51 features (running kinematic and kinetic variables)	Several feature extraction methods → AdaBoost (as part of the classifier)	AdaBoost (84.7–100%)	Differences between gender, shod/barefoot running, and runners with and without PFP
[47]	40	505 features (5 running kinematic waveforms)	PCA (the first 3 PCs/waveform)	–	Differences between female runners with previous ITBS and female healthy runners
[19]	72	902 features (9 running kinematic waveforms + 2 clinical variables)	PCA (only kinematic waveforms) → SFS (the best 2 PCs)	LDA (78.1%)	Prediction of the response to exercise treatment for patients with PFP
[20]	98	604 features (6 walking kinematic waveforms + 4 clinical variables)	PCA (only kinematic waveforms) → SFS (the best 6 PCs and 1 clinical variable)	LDA (85.4%)	Prediction of the response to exercise treatment for patients with knee OA
[45]	200	4 features (running kinematic variables) or 100 features (a running kinematic waveform)	PCA, Kernel PCA (the first 7 or 10 PCs)	–	Gender- and age-differences in healthy runners
[28]	11	3939 features (39 running marker position waveforms)	PCA, PCA with SVM, ICA → MR	–	Differences between movements resulting from wearing shoes with different midsoles
[14]	121	900 features (9 running kinematic waveforms)	PCA (the first 4 PCs/waveform)	HCA	Defining distinct groups of healthy runners and to investigate the practical implications of clustering healthy subjects
[67]	88	3636 features (36 running marker position waveforms)	SOFM	*k*-Means	Defining functional groups of runners and to understand whether the defined groups required group-specific footwear features

Another input feature method involves a position matrix, which is based on the marker position data [15, 16]. The size of an initial feature vector based on 3D marker position is usually larger than an initial input vector based on joint angles. However, more data does not necessarily mean more useful information since it may contain more ambiguous or erroneous data such as soft tissue movement artefact. While similar results obtained from the same statistical group and supervised learning classification approaches were found in a comparison between two previous studies: one with joint angle data [2] and the other with marker position data [25], the clinical relevance of marker position data is questionable. Thus, joint kinematic angles are recommended to use as initial input features to improve the clinical relevance of the results.

Due to the fact that some dimensionality reduction and machine learning methods require an adequate number of samples to obtain stable results, the dimensionality of the initial input features used in the analysis should be carefully chosen. For example, Barrett and Kline [26] recommended that the number of subjects should be at least 50 for a PCA approach. Unfortunately, previous research involving a PCA approach have utilized small cohorts of subjects (*n* = 10–30) [17, 18]. Thus, to minimize the high-dimensionality of the data, big data in terms of big volume (i.e., a large cohort of subjects) are needed to re-evaluate the proposed model and find a consensus among various models or studies.

## Dimensionality Reduction

To analyze a large number of biomechanical gait variables involving many joints and planes of motion, techniques to retain information that is important for class discrimination and discard that which is irrelevant are necessary. To determine which data should be retained and which can be discarded, dimensionality reduction has been used. Specifically, dimensionality reduction can be defined as the process of reducing data size or the number of initial input features, and this approach is expected to be able to operate effectively in big data analytics. Although the size of the data in most current studies might be efficiently processed using traditional multivariate and machine learning methods in a single high performance computer, to process larger-scale and more complex data in future studies, it is necessary to re-design and change the way the traditional multivariate and machine learning methods are computed. One way is to use more efficient data analysis methods that can accelerate the computation time or to reduce the memory cost for the analysis. Another way is to create methods that are capable of analyzing big data by modifying traditional methods that work on a parallel computing environment or even to develop new methods that work naturally on a parallel computing or a cloud computing environment. It should be noted that these approaches not only reduce the computational cost, but can also possibly improve classification accuracy by reducing the noise and improving the clinical relevance of the results by selecting more interpretable features.

### Feature Selection

Instead of choosing the appropriate features based on investigator’s background knowledge, feature selection approaches using data science methods return the best subset of the initial input features. These methods aim to maximize a features’ relevancy and/or minimize a features’ redundancy using a combination of an objective function with a search strategy.

#### Objective Function

There are two types of measures to score features: wrapper and filter. Wrapper methods use a specific classifier with a cross-validation method to provide a score, or a classification rate, for each feature subset. Measures applied using this method include a linear discriminant analysis (LDA) [19, 20] and SVM [27]. Although wrapper methods typically provide the best performing feature set for a specific classifier (since the characteristics of the selected features match well with the characteristics of the classifier), there are no guarantees that this feature subset will perform best for other classifiers. Moreover, the computational cost of wrapper methods is higher than filter methods and to perform wrapper methods for big data, extensive computational time to search the best feature subset is necessary, thus parallelized implementations of cross-validation may be ideal.

In contrast, filter methods use interclass distance/similarity, correlation, or information-theoretic measures, to score features. Measures applied in this field include the effect size (i.e., Cohen’s *d* [2, 3, 13, 28]) and the outcomes of statistical tests (i.e., *t*-test). Although mutual information has not been applied in this field yet [29], this measure offers potential value when initial features consist of both categorical data (e.g., demographic data and clinical data) and continuous/discrete data (e.g., kinematic data). While filter methods generally provide lower prediction performance than wrapper methods, the selected feature subset is more generalisable and thus more useful for understanding the associations between features. Filter methods can also be used as a pre-processing step [13] for feature extraction, allowing for more stable results when the dimensionality of initial input is high. Filter methods are also less computationally expensive and often easier to implement than multi-level wrapper functions.

#### Search Strategy

The simplest and most popular search approach is to apply an objective function, such as the filter method, to each feature individually to determine the relevance of the feature related to the target class (or the classification variable), and then select the top-ranked (or the best) features according to these outcome scores. This approach is typically called a univariate feature selection or maximum-relevance (MR) selection method. Improvements in classification and interpretation have been observed consistently among previous studies that have applied this approach [2, 3, 24]. The top-ranked features, however, could be correlated among themselves and have different robustness ability. Therefore, features selected according to their discriminative powers do not guarantee a better feature set. Thus, one solution is to include minimum redundancy criteria (i.e., features should maximally dissimilar to each other) such as implemented in a minimum redundancy–maximum relevance algorithm [29].

Another popular search approach in the data science field is sequential feature selection (SFS) algorithms. This family of “greedy” search algorithms creates a subset of features by adding or removing one feature at a time, based on a score obtained from a wrapper method, and then chooses the subset that best increases the classification accuracy while also minimizing the feature subset size. This algorithm has achieved good classification performance to select a subset of discrete variables in several investigations. For example, Fukuchi et al. [27] applied a sequential forward selection to search for the best subset of features among thirty-one kinematic variables in identifying age-related differences in running gait biomechanics. This algorithm consistently selected the knee flexion excursion angle (a decrease in the angle among older runners), which has been usually reported in the literature using classical inferential statistics [30, 31]. The results can be used to better understand the greater incidence of injuries among older walkers and runners which might be due to age-related changes in gait patterns [32]. In addition, Watari et al. [19] and Kobsar et al. [20] used this approach to search the best feature subset from a mixed group of baseline kinematic, clinical, and patient-reported outcome variables, achieving a good performance (78–85%) in predicting treatment outcome in patients with patellofemoral pain (PFP) and knee osteoarthritis (OA), respectively. It is important to note that PFP is the most common injury among runners [33] and PFP has been associated with the development of knee OA since between 25 and 35% of patients report little-to-no improvements in their symptoms following treatment. The findings of both studies support the use of feature selection of baseline kinematic variables to predict the treatment outcome for patients with PFP and knee OA, which is a significant step towards a method to aid clinicians by providing evidence-informed decisions regarding optimal treatment strategies for patients.

Unfortunately, the SFS and related algorithms use an incremental greedy strategy for feature selection and tend to become trapped in local minima, particularly when dimensionality is very high. To deal with higher-dimensional data in future studies, algorithms need to incorporate randomness into their search procedure to escape local minima. Some potential and well-known population-based metaheuristic methods are genetic algorithm (GA) [34], ant colony optimization [35], particle swarm optimization [36], and harmony search [37]. To our knowledge, no prior studies have applied these advanced search techniques in walking and/or running biomechanical research. These search techniques have also been developed to work in parallel computing and can be used for big data analytics such as parallel computing version of GA [38].

Finally, feature selection algorithms can be also embedded within learning algorithms such as decision tree and regularized trees [39]. Specifically, an AdaBoost, Adaptive Boosting, algorithm improves the performance of other learning algorithms (as called weak learners) by combining their outputs into a weighted sum/vote (a strong classifier) that represents the final output/decision. Although embedded methods can reduce the cost of exploring larger search spaces, they could still yield poor generalization performance (like the wrapper methods). For example, Eskofier et al. [40] applied AdaBoost with decision trees as weak classifiers, to select the best feature subsets from kinematic and kinetic running data. Good classification results (84–100% accuracy) were found for all experiments: gender, shod/barefoot, and injury groups. However, while this technique appears promising, the aforementioned study [40] involved a relatively small data set (*n* = 12) so future research involving much larger amounts of data should be employed.

### Feature Extraction

Instead of selecting a subset of initial input features, feature extraction approaches transform a set of values in an original feature space into a set of values in a new lower-dimensional space. In other words, feature projection or feature transformation methods attempt to determine the best combination of the initial input features. It is important to note that gait biomechanical features can be extracted in time domain, frequency domain, and time–frequency representation [23, 41–43]. Features can be also based on either linear or non-linear analysis as well as based on either supervised- or unsupervised-learning approach [28, 41, 42, 44–46].

#### Linear Unsupervised Feature Extraction

Investigations of walking and running gait biomechanics are mostly based on the PCA method, which involve the common idea of dimensionality reduction to create a new set of uncorrelated variables, known as principal components (PCs). The PCs are linear combinations of the original possibly correlated variables, and this method has demonstrated classification accuracies between 80 and 100% in identifying differences in running gait patterns between different cohorts [2, 3, 15, 16, 24, 25]. Often, however, researchers account for nearly all of the between-group variance from the original features using the first few, or lower-order, PCs [47, 48]. In these analyses, only a set of the first few PCs are retained, whereas the remaining set of intermediate- and higher-order PCs are ignored. Unfortunately, there is no guarantee that the difference between the groups of interest will be in the direction of the first few, or high-variance, PCs and focusing only on first PCs, which are associated with the most dominant movement patterns (e.g. gender- and age-related differences, or differences between runners with and without ITBS [3, 25, 47]), may exclude important information necessary for class separability between groups of interest. In contrast, intermediate- and higher-order PCs are often associated with subtle movement patterns. For example, Phinyomark et al. [3] showed that these higher-order PCs could be potentially useful in monitoring the progress of treatment outcomes for injured patients or athletes and thereby predicting rehabilitation success or risk of injury reoccurrence. The higher-order PCs showing differences between baseline and following a 6-week muscle strengthening program for runners with PFP also exhibited a moderate and significant linear correlation with clinical outcome data [3] while the first few PCs have no significant correlation. In addition, Nigg et al. [25] used the higher-order PCs to investigate the effects of shoe midsole hardness on running kinematics.

While meaningful features derived from the PCA methods can be identified, there are several ways to interpret the biomechanical and clinical meaning of these features. Using the visual inspection of the shape of the PC loading vector, several PC feature types can be defined such as magnitude features, difference operator features, and phase shift features [23, 49]. For instance, features extracted from the first PC generally capture the magnitude information and thus show strong correlation with discrete variables in the magnitude group (such as joint angles at touchdown and toe-off) [23]. Traditional solutions for waveform analysis are to compare the differences between two representative original gait waveforms chosen from some quantiles of the PC-scores/features for the PC of interest [48]. However, the observed differences between raw gait waveforms generally involve not only the biomechanical features extracted from the PC of interest but also the remaining information captured by other unselected PCs. To isolate the inter-subject variance explained by the PCs of interest, we can use reconstructed gait waveforms by reversing a single [49] or a set [50] of interested PCs back to the original space, instead of raw gait waveforms. For instance, a meaning set of 156 PCs was used to reconstruct fourteen different gait waveforms and these reconstructed waveforms can be used to illustrate and identify subtle systematic differences in gait movement patterns for runners with high and low weekly running mileage [50].

For interpreting PCs computed from discrete variables, the correlation (Pearson’s *r*) between initial input features and PC features is computed to provide the information shared between them [51]. The variables with the highest correlation (maximum value of *r*) or strong correlations (for example, *r* > 0.67 [2]) with each chosen PC have been used to interpret its meaning. To aid in interpreting intermediate- and higher-order PCs, which generally demonstrate weak correlations with input features, Phinyomark et al. [3] proposed two different approaches: (1) the percentage of sum of the squared correlation coefficients between each of the PCs and all the original variables in the interested joint and/or plane of motion, and (2) the percentage of sum of the squared correlation coefficients between each of the original variables and all the PCs that are needed to yield the maximum classification performance between groups of interest. These approaches have been successful in interpreting the biomechanical meaning of PCs in several clinical research questions of interest (e.g., differences in walking gait patterns between subjects with and without knee OA [24]).

The standard PCA method often encounters difficulties when data are non-linear or non-Gaussian distributed. Although some kinematic variables of interest are normally distributed for both injured and non-injured runners [2], based on our preliminary work, the probability density of running gait kinematics in transverse plane kinematics may not be Gaussian and thus non-linear. Therefore, feature projection methods with non-Gaussian and/or non-linearity assumptions should be investigated. Specifically, since standard PCA considers only the second-order statistics (i.e., co-variance), this method relies heavily upon the Gaussian features. In contrast with PCA, independent component analysis (ICA) employs higher-order statistics and exploits inherently non-Gaussian features of the data.

#### Non-linear Unsupervised Feature Extraction

Data transformation methods can also be employed in a non-linear manner and the standard PCA method can be extended to model data distributions in high-dimensional space by using a kernel technique called “kernel PCA”. The performance of this non-linear feature projection-based method for identifying differences in running gait patterns between different cohorts increases as compared to the linear PCA [45]. Unfortunately, the computational cost of these non-linear methods is high in comparison to the linear methods, and may cause a problem in big data analysis. One solution is to create a linear-nonlinear feature projection by using a linear projection-based method (e.g. PCA) to reduce dimensionality of the initial input features and then transforming the reduced and projected features into a new high-class-separability feature space using nonlinear mapping by a nonlinear projection-based method (e.g., self-organizing feature maps (SOFM)) [46].

Fractal analysis is used to assess fractal, or self-similarity, characteristics of data by determining the fractal dimension of data. Instead of considering variability to be random [52], fractal analysis considers variability as long-term correlations. These methods have been widely used to study many biomedical data [52–56] involving the complex fluctuations in gait patterns or dynamic stability (e.g. gait variability: stride interval variability and the variability of the center-of-mass) [41, 57]. We can use information obtained from these studies to quantify human gait movement and its changes with aging and disease and to identify changes in biomechanics after a therapeutic intervention/rehabilitation protocol [30]. There are numerous definitions of fractal dimension, and several potential techniques of fractal analysis include detrended fluctuation analysis (DFA), the maximum Lyapunov exponent (MLE), critical exponent analysis and variance fractal dimension. Specifically, DFA can be used to quantify stride interval dynamics while MLE quantifies local dynamic stability [58, 59]. Unfortunately, to apply fractal analysis very long time-series are required (i.e., hundreds to thousands of gait cycles are necessary). On the other hand, in clinical practice, 3D gait kinematics and EMG are measured during a short period, particularly for subjects with certain pathologies [41].

#### Supervised Feature Extraction

A combination of SVM and ICA as a supervised-unsupervised feature projection has shown better performance in classifying kinematic running data with two different shoe conditions as compared to the linear PCA method [28]. These supervised feature projection methods not only reduce the dimensionality of the initial input features but they also improve class separability. Other supervised methods of dimensionality reduction should be investigated in future studies such as LDA and its extended versions (e.g., uncorrelated LDA, orthogonal LDA, orthogonal fuzzy neighborhood discriminant analysis, generalized discriminant analysis, LDA via QR-decomposition, and a combination of LDA, fuzzy logic and the differential evolution optimization technique) [44].

## Machine Learning

### Supervised Machine Learning

After a final feature vector is created, either a supervised or an unsupervised learning approach is needed to perform the classification or clustering. For classification, the most popular supervised learning method in this field is the SVM classifier [2–4, 15, 16, 22–25, 27, 28, 50, 60–62]. SVM builds a model that predicts whether a new subject best fits in one category or the other (i.e., a binary linear classifier) and we can also extend it for multi-class classification by reducing the single multiclass problem into multiple binary classification problems (i.e., building binary classifiers which discriminate either between one of the class labels and the rest (*one*-*versus*-*all*) or between every pairs of classes (*one*-*versus*-*one*). As one example, although SVM can efficiently perform a nonlinear classification, Fukuchi et al. [27] compared the classification performance of several linear and nonlinear kernels and found that a linear kernel exhibits better performance in identifying age-related differences in running gait biomechanics as compared to non-linear kernels involving polynomial and Gaussian radial basis function kernels. For walking gait biomechanics, the best SVM model has been also found to be the linear SVM (e.g., to detect age-related changes in walking gait patterns [60], to identify walking gait pattern of patients with PFP [61], and to identify walking gait pattern of patients with OA as well predicting gait improvement after knee replacement surgery [62]). Non-linear kernels have also been found to overfit the data as compared to the linear model [62].

Hence, for a more robust model, an LDA classifier is recommended [63]. This classifier has been able to successfully predict individual treatment success to an exercise intervention in knee OA patients using a combination of baseline patient-reported outcome measures and kinematic variables [19, 20]. Lee et al. [64] shows that LDA could be applied successfully to discriminate walking gait patterns with an unknow load condition. Further, to estimate the generalisation capability of a given classifier, either a leave-one-out cross-validation method (for small sample size) or a *k*-fold cross-validation method (for large sample size) should be performed. For a ten-fold cross validation, as an example, input features for a classifier are randomly partitioned into 10 equally sized subgroups and a single subgroup is retained as testing data while the remaining nine subgroups are used as training data for the classification model. The cross-validation process is then repeated 10 times, and a single classification rate, or confidence interval, is computed from the 10 iterations.

### Unsupervised Machine Learning

In contrast, when target classes are not available, naïve clustering or cluster analysis is needed. In brief, a cluster analysis method is used to measure the similarities in gait data within a heterogeneous set of individuals and then group sets of similar observations, or patterns, are created. For example, Phinyomark et al. [14] applied hierarchical cluster analysis (HCA) to determine if running gait patterns for 121 healthy subjects could be classified into homogeneous subgroups using 3D kinematic data. Two distinct running gait patterns were found with the main between-group differences occurring in frontal and sagittal plane knee angles, independent of age, height, weight, and running speed. When these two groups were separately compared to a large cohort of runners experiencing PFP, the most common running related injury, two different outcomes were shown for differences in peak knee abduction angles. This technique has been also applied on walking gait patterns of normal subjects, and the results show there are similarities between walking and running studies. Specifically, Simonson and Alkjær [65] found significant differences between two sub-groups of walkers based on sagittal plane knee kinematics while Mezghani et al. [66] found significant differences between four sub-groups of walkers based on frontal plane knee kinematics. Considering these similarities, the primary differences in walking and running gait patterns of healthy subjects can be observed in frontal and sagittal plan knee joint angles. In a similar manner, Hoerzer et al. [67] use a *k*-means clustering algorithm to define eight functional groups based on their distinctive gait patterns to understand whether the defined groups required group-specific footwear features. It should be noted that *k* stands for number of clusters and is an input parameter for the algorithm. Thus, an inappropriate choice of *k* may yield poor results. These results suggest that variability observed in gait patterns in a sample of subjects could be the result of different gait strategies represented within the sample, rather than because of another discriminating factor (i.e., injury status). Further, previous studies have used the HCA approach to identify distinct walking gait patterns among patients (e.g., cerebral palsy [68], chronic stroke [69], and Charcot-Marie-Tooth disease [70]) and thereby clinicians can use these defined sub-groups to assess the effects of different treatments via tracking changes in specific gait patterns in each group of patients.

## Topological Data Analysis

Recently, a number of techniques rooted in algebraic topology have been proposed as novel tools for data science analysis and pattern recognition. The fundamentally new character of these tools, collectively referred to as TDA or topological data analysis, stems from abandoning the standard measures between data points (or nodes, in the case of similarity networks) as the fundamental building block, and focusing on extracting and understanding the “shape” of data at the mesoscopic scale. In doing so, this method allows for the extraction of relevant insights from complex and unstructured data without the need to rely on specific models or hypotheses. In addition, topology is written in the language of set theory and provides a natural candidate to approach, in a formal way, higher-order dimensional patterns [71].

Algebraic topology techniques have been recently used with success in biological and neurological contexts and play a key role in understanding of complex systems in a wide range of fields by extracting useful information from big datasets [72–76], thanks to their ability to capture macro- and meso-scale coordination from local information [77]. We can now begin to understand that geometry and topology make useful contributions to the analysis of data and can be leveraged to enhance machine-learning approaches by providing new, non-trivial features. While geometry can be intuitively regarded as the study of distance functions, topology captures robust information about shapes in high-dimensional spaces and is therefore invariant under deformations, rotations and many of the transformations that would corrupt geometrical features. In this paper, two major TDA techniques are presented, which can be considered as potential big data analytics for running gait analysis: (1) topological simplification, as exemplified by the Mapper algorithm [78–80], and (2) persistent homology [81, 82].

### Topological Simplification

**Fig. 3:**
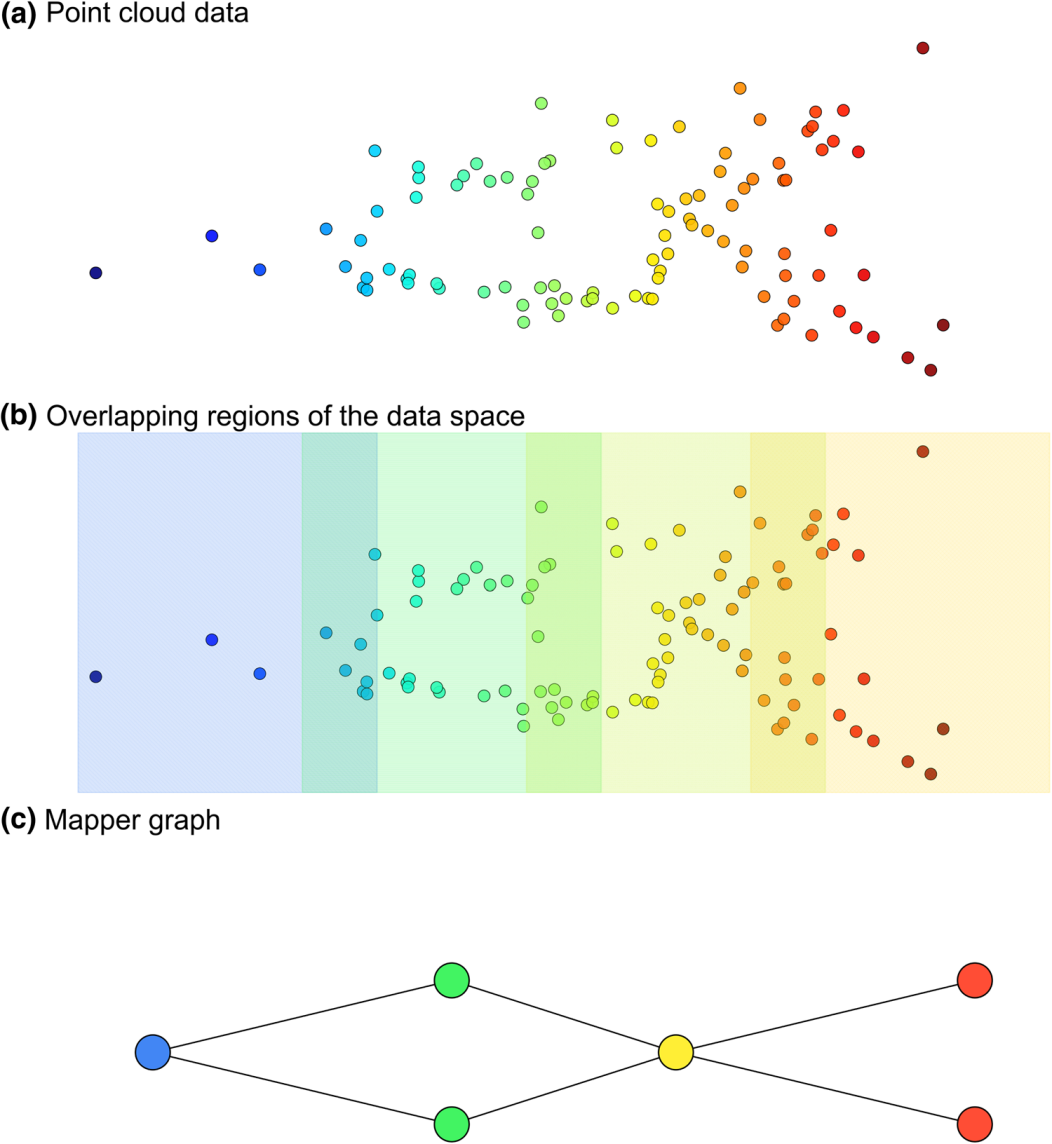
Pipeline of topological simplification (the Mapper algorithm)

Topological simplification refers to a set of techniques that can extract a topological backbone from an unstructured data cloud. The most well-known technique was originally introduced by Singh et al. [78] and has found many applications both in research and in industry [79, 80]. For example, if a metrical or similarity structure is available, it is possible to produce controlled simplications of the structure by means of a series of local clusterings in overlapping regions of the data space, and then linking together clusters that share common data points. This is the basis of the Mapper algorithm (Fig. 3). Specifically, this algorithm relies on two main modules: a filter function and a clustering protocol. The filter function is used to define a set of overlapping regions that slice the point cloud data. Within each region, the points are then clustered according to the chosen clustering protocol. Because of the overlap between regions, some clusters in adjacent regions will have common points. The Mapper graph is then constructed considering the clusters as nodes and adding an edge between two clusters whenever they have a non-empty intersection, hence yielding a simplified skeleton-like representation of the original point cloud data. This method is based on topological ideas in the sense that the described construction tends to preserve the notion of nearness in the data cloud, but discard the effects of large distances which often carry little meaning or are scarcely reliable in applications.

The output of this topological simplification approach has been used in several cases as cluster analysis to extract non-trivial qualitative information from large datasets that was hard to discern when studying the dataset globally. Nicolau et al. [79], for instance, detected a previously unknown subtype of breast cancer characterized by a greatly reduced mortality while Lum et al. [80] and Nielson et al. [83] identified a number of new subgroups in datasets as diverse as genomic data, NBA players, and spinal cord injuries.

More interestingly, it is possible to use the output of the algorithm to engineer relevant features for further classifications as feature selection. Guo and Banerjee [84] selected, topologically, a set of key process variables in a manufacturing pipeline that affected the final yield and reduced the monitoring and control costs. Specifically, after the Mapper graph is constructed, we can define fundamental and interesting subgroups (in which their shapes persist over a large-scale change of the resolution parameters), and then statistical tests (like *t*-test or ANOVA) can be performed among the subgroups to select the best discriminatory features.

The aforementioned approaches have shown potential, and are therefore good candidates as a way to build simplicial complexes used to compute persistent homology that convey local summaries of the dataset’s features. However, one of the main limitations of Mapper is that it requires a specific choice of scales, in the definitions of the bins, of their overlap, and also in the choice of the underlying filter function. This limitation is typically dealt with by swiping across a range of parameters and ensuring that the result is stable, but there is no formal way to choose the optimal parameter set. This problem is a common one for tools that are based on set theoretic concepts, because in most applications the sets need to be defined, and that entails a choice of parameters. Persistent homology, however, turns this limitation upside down by embedding this scaling problem in its definition.

### Persistent Homology

**Fig. 4:**
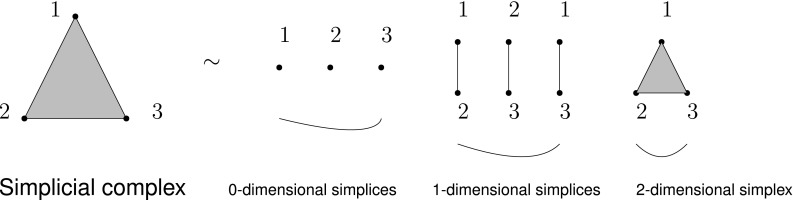
Simplicial complex and 0-, 1-, and 2-dimensional simplices

Persistent homology studies the shape of data across a range of scales [81]. It does this by studying the homology of a point cloud, i.e., the patterns of holes in all dimensions that define the multidimensional shape of a dataset. To do this, one must produce a simplicial approximation to the dataset. Specifically, a simplicial complex is a topological space constructed by the simplices where simplices are points, lines, triangles, and their *n*-dimensional counterparts. We can obtain *a simplicial complex* from a graph where *simplices* are nodes (in dimension zero), edges (dimension one), full triangles (dimension two), tetrahedron (dimension three), and so on (Fig. 4). It needs to respect a consistency rule requiring that the intersection of any pair of simplices in a simplicial complex is another simplex in the same simplicial complex. The generality of their definition allows simplicial complexes to describe relational patterns that include interactions between any number of elements, potentially also in different dimensions.

**Fig. 5:**
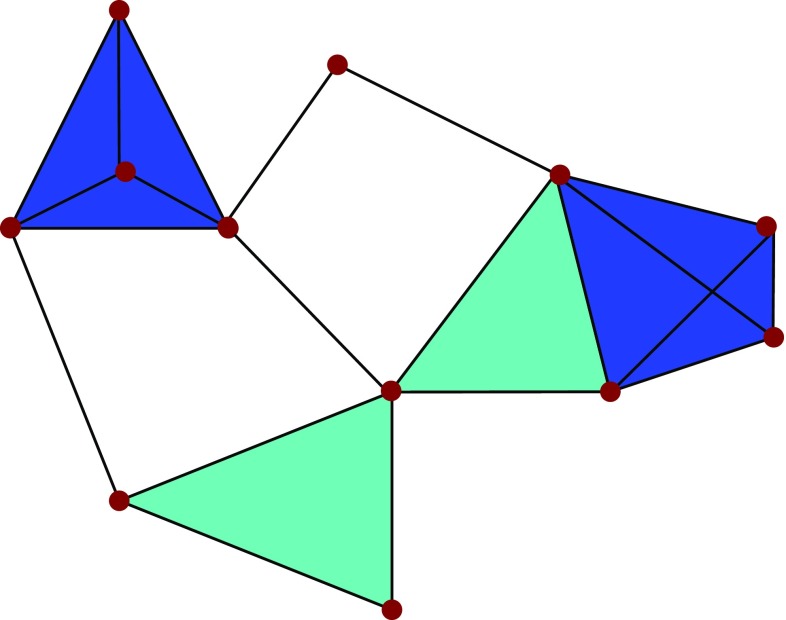
Clique complex where cliques of size one, two, three, and four are shown as small *red disks*, *black line* segments, *light blue triangles*, and *dark blue tetrahedral*, respectively

There are two kinds of inputs from where we are able to construct a simplicial complex. First, simplicial complexes can build from networks (e.g. real-world complex networks [85, 86], spreading processes [87], and structural and functional brain networks [73, 76]), via *clique complexes* (Fig. 5). A clique complex from a network is formed by the sets of vertices in the network’s cliques. A clique is a subset of vertices such that they induce a complete subgraph. That is, every two distinct vertices in the clique are adjacent. Converting a graph to a simplicial complex reveals mesoscopic organizational structure that was not appreciable at the network level, thanks to the non-locality of the topological invariants of the simplicial complex.

**Fig. 6:**
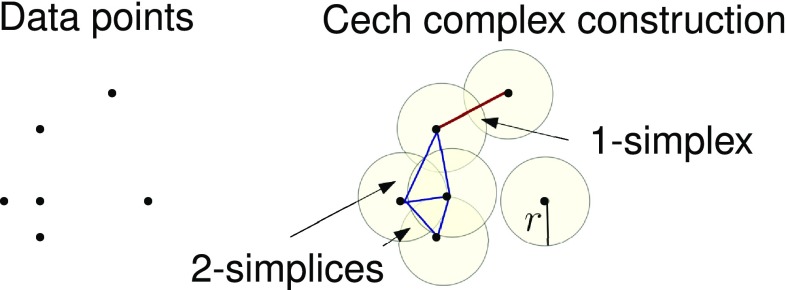
Cech complex where 1-simplex and 2-simplices are shown as *red line* and *blue full triangles*, respectively

Second, if a set of data points with metrical information is given, it is possible to construct a simplicial complex by leveraging the metrical structure via Cech or Rips-Vietoris simplicial complexes [82], i.e., simplicial complexes whose simplices are defined in terms of overlapping neighborhoods of the data points. When the data comes with a metric, the typical way of doing this is by constructing *a Cech complex* (Fig. 6): one considers the neighbourhood of radius *r* of each point; whenever two neighbourhoods overlap one adds the 1-simplex formed by the corresponding two points, when three neighbourhoods overlap one adds the corresponding 2-simplex, and so on. The obvious problem here is that there is no a priori way to choose the value of the radius *r*.

**Fig. 7:**
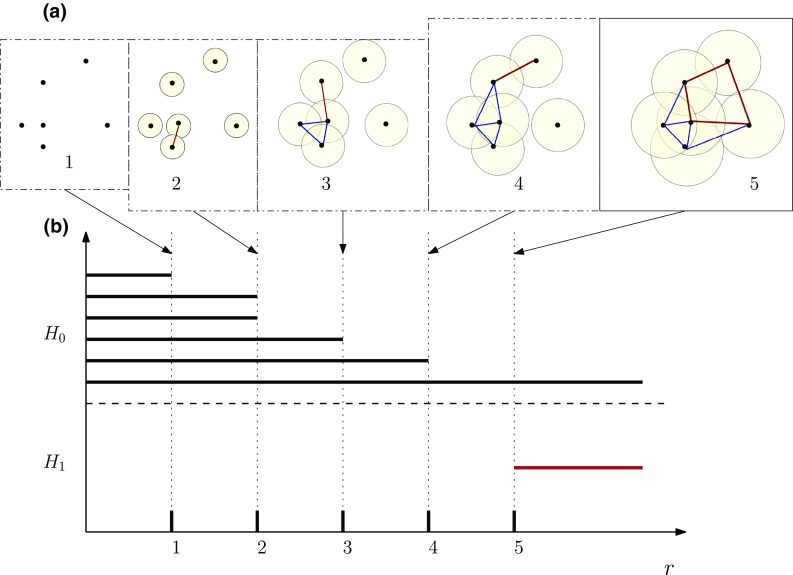
**a** Cech complex where a fixed set of points (step 1) can be transformed into different Cech complexes based on a proximity parameter *r*. **b** Barcode of H_0_ (the number of connected components) and H_1_ (the number of cycles) according to the evolution of proximity parameter *r*

Persistent homology solves the problem of select a radius *r* through focusing, by design, on the features that live across intervals of values and assigns an importance to them proportional to the length of such intervals (Fig. 7a) with the obvious implication that features surviving across many scales are more meaningful than those that live only for short intervals. This setup appears deceptively simple, but it has been proven to be extremely powerful, versatile, and robust to noise, both at the theoretical and applied level [88]. Indeed, the range of applications has been steadily growing over the recent few years, spanning very diverse fields, e.g. viral evolution [72], brain imaging [73, 76], sensor coverage [86], vision [89], population genetics [90], and dynamical systems [91, 92]. A few applications also show that using persistent homology information to enhance classification or clustering schemes can indeed be very powerful, and can highlight non-trivial groups that were previously undiscernible.

The reason of the efficacy of homological information in enhancing clustering scheme lies in the fact that topological information is shaped by mesoscopic properties of the data that require the coordination of a large number of points and cannot be described only by local properties. In this way homology captures hidden correlations in the data that are hard to describe using standard statistical tools.

### Topological Methods for Machine Learning in Gait Analysis

Machine learning approaches involving dimensionality reduction (see Sect. 5) and learning algorithms (see Sect. 6) still remain largely unknown in running biomechanical research and their full potential has not been yet to be realised. Regardless, TDA methods would largely benefit running gait analysis and should also be investigated in future studies. As one example, the analysis of running gait data could involve the first TDA technique (Sect. 7.1) as cluster analysis and/or feature selection while we can apply the second TDA technique (Sect. 7.2) as feature extraction. Thus, we propose that novel TDA methods can be used either *instead of* or *together with* existing machine learning methods.

For instance, applying TDA as a cluster analysis method could overcome the aforementioned disadvantages of techniques such as HCA which is not very robust to outliers, or the *k*-mean clustering method that requires the number of clusters to be specified in advance. Furthermore, in biomechanical gait analysis it is widely known that variability of the data is very high which involves within-trial, between-trial, intra-subject and inter-subject variabilities, including several kinds of noise. Vardaxis et al. [93] showed that the distinct groups of normal subjects identified by HCA applied to 3D gait data remained unchanged only up to the removal of three subjects. In contrast, TDA methods describe the global topological structure of the point cloud data rather than their local geometric behavior, and are thereby robust to missing data and small errors, making TDA a good candidate to cluster running gait patterns with large volumes of sparse data. Similar to other manifold learning algorithms [94], TDA methods also allow the researcher to unfold and capture non-linear structures that are not well described by linear separation algorithms (e.g. the Swiss roll). Generally, biomechanical gait variables interact with one another in a complex non-linear fashion due to the intrinsic non-linear dynamics of human movement. In addition, thanks to the local nature of the clustering, TDA methods naturally provide a separation of the global clustering problem into a set of many smaller problems, which are immediately amenable to parallelization. In this way, Mapper and other related algorithms can address problems that utilize large, high-dimensional data sets.

It is also possible to couple TDA with other machine learning approaches to gain deeper insights into running gait data. For example, the use of persistent homology as a novel feature extraction method, combined with a classifier (e.g. SVM), could create a pattern recognition system to study the subtle changes or differences in gait patterns. There are several techniques that could be applied to extract meaningful features [e.g., Betti numbers and barcodes [Fig. 7b)] from gait data. For example, persistent homology TDA techniques may be able to deal with biomechanical data collected during walking or running, which has a quasi-periodic temporal dependence. Using techniques such as those proposed by Pokorny et al. [74], researchers can enhance trajectory classification in a 3D space with different obstructions by studying the relative homology of the space once the starting and ending points of the trajectories were identified. Specifically, the persistent homology TDA techniques naturally describe periodic or recurrent behaviours—as well as departures from these—and are therefore suitable to extract robust trajectories in high dimensional spaces, which can be then directly interpreted as features of typical and atypical gait datasets. Finally, large datasets can be effectively subdivided and studied in parallel via chunk homology, which has a cubic complexity in the number of chunks or the maximal size of the chunks. The global homological structure is then recovered easily by collating the results of the mutually independent (in this case, acyclic) chunks [95]. Hence, these techniques can be applied to data with high-dimensionality, temporal dependence, high variability, and nonlinear relationships, which are prominent challenges in the analysis of gait data [41, 42].

## Conclusions

In recent years, technological advances now provide researchers with large amounts of data, which can be explored for meaningful patterns. For instance, the 3D GAIT system is an automated 3D biomechanical gait data collection system wherein all data are transferred to a central research database. While traditional data analytics cannot handle these large volumes of data, various “big data” statistical methods should be investigated and developed. All of the methods discussed in this paper show promise, provide inspiration for future work, and demonstrate the potential of using data science methods in running gait biomechanics research.

## References

[1] Louw M, Deary C (2014). The biomechanical variables involved in the aetiology of iliotibial band syndrome in distance runners—A systematic review of the literature. Physical Therapy in Sport.

[2] Phinyomark A, Hettinga BA, Osis ST, Ferber R (2014). Gender and age-related differences in bilateral lower extremity mechanics during treadmill running. PLoS ONE.

[3] Phinyomark A, Hettinga BA, Osis ST, Ferber R (2015). Do intermediate- and higher-order principal components contain useful information to detect subtle changes in lower extremity biomechanics during running?. Human Movement Science.

[4] Fukuchi, R. K., Stirling, L., & Ferber, R. (2012). Designing training sample size for support vector machines based on kinematic gait data. In *Proceedings of 36th Annual Meeting of the American Society of Biomechanics*.

[5] Tsai CW, Lai CF, Chao HC, Vasilakos AV (2015). Big data analytics: A survey. Journal of Big Data.

[6] Herland M, Khoshgoftaar TM, Wald R (2014). A review of data mining using big data in health informatics. Journal of Big Data.

[7] Demchemko, Y., Grosso, P., de Laat, C., & Membrey, P. (2013). Addressing big data challenges in scientific data infrastructure. In *Proceedings of IEEE 4th International Conference on Cloud Computing Technology and Science*, 614–617.

[8] Söderkvist I, Wedin PA (1993). Determining the movements of the skeleton using well-configured markers. Journal of Biomechanics.

[9] Cole GK, Nigg BM, Ronsky JL, Yeadon MR (1993). Application of the joint coordinate system to three-dimensional joint attitude and movement representation: A standardization proposal. Journal of Biomechanical Engineering.

[10] Osis ST, Hettinga BA, Leitch J, Ferber R (2014). Predicting timing of foot strike during running, independent of striking technique, using principal component analysis of joint angles. Journal of Biomechanics.

[11] Osis, S.T., Hettinga, B. A., & Ferber, R. (2015). Predicting timing of foot strike for treadmill walking and running with a principal component model of gait. In *Proceedings of XXV Congress of the International Society of Biomechanics*.

[12] Laney, D. (2001). 3D data management: Controlling data volume, velocity, and variety. META Group. Retrived October 14, 2016, from http://blogs.gartner.com/doug-laney/files/2012/01/ad949-3D-Data-Management-Controlling-Data-Volume-Velocity-and-Variety.pdf.

[13] Phinyomark A, Osis ST, Hettinga BA, Leigh R, Ferber R (2015). Gender differences in gait kinematics in runners with iliotibial band syndrome. Scandinavian Journal of Medicine and Science in Sports.

[14] Phinyomark A, Osis ST, Hettinga BA, Ferber R (2015). Kinematic gait patterns in healthy runners: A hierarchical cluster analysis. Journal of Biomechanics.

[15] Eskofier BM, Federolf P, Kugler PF, Nigg BM (2013). Marker-based classification of young-elderly gait pattern differences via direct PCA feature extraction and SVMs. Computer Methods in Biomechanics and Biomedical Engineering.

[16] Maurer C, Federolf P, von Tscharner V, Stirling L, Nigg BM (2012). Discrimination of gender-, speed-, and shoe-dependent movement patterns in runners using full-body kinematics. Gait & Posture.

[17] Maurer C, von Tscharner V, Samsom M, Baltich J, Nigg BM (2013). Extraction of basic movement from whole-body movement, based on gait variability. Physiological Reports.

[18] Federolf P, Tecante K, Nigg B (2012). A holistic approach to study the temporal variability in gait. Gait & Posture.

[19] Watari R, Kobsar D, Phinyomark A, Osis ST, Ferber R (2016). Determination of patellofemoral pain sub-groups and development of a method for predicting treatment outcome using running gait kinematics. Clinical Biomechanics.

[20] Kobsar D, Osis ST, Hettinga BA, Ferber R (2015). Gait biomechanics and patient-reported function as predictors of response to a hip strengthening exercise intervention in patients with knee osteoarthritis. PLoS ONE.

[21] Batista GEAPA, Monard MC, Abraham A, Ruiz-Del-Solar J, Koppen M (2002). A study of *k*-nearest neighbour as an imputation method. Soft computing systems: Design, management and applications.

[22] Lai DTH, Levinger P, Begg RK, Gilleard WL, Palaniswami M (2009). Automatic recognition of gait patterns exhibiting patellofemoral pain syndrome using a support vector machine approach. IEEE Transactions on Information Technology in Biomedicine.

[23] Phinyomark, A., Osis, S. T., Kobsar, D., Hettinga, B. A., Leigh, R., & Ferber, R. (2016). Biomechanical features of running gait data associated with iliotibial band syndrome: discrete variables versus principal component analysis. In E. Kyriacou, S. Christofides, C. S. Pattichis (Eds.), *XIV Mediterranean Conference on Medical and Biological Engineering and Computing 2016* (pp. 580–585). Springer.

[24] Phinyomark A, Osis ST, Hettinga BA, Kobsar D, Ferber R (2015). Gender differences in gait kinematics for patients with knee osteoarthritis. BMC Musculoskeletal Disorders.

[25] Nigg BM, Baltich J, Maurer C, Federolf P (2012). Shoe midsole hardness, sex and age effects on lower extremity kinematics during running. Journal of Biomechanics.

[26] Barrett PT, Kline P (1981). The observation to variable ratio in factor analysis. Personality Study and Group Behaviour.

[27] Fukuchi RK, Eskofier BM, Duarte M, Ferber R (2011). Support vector machines for detecting age-related changes in running kinematics. Journal of Biomechanics.

[28] Von Tscharner V, Enders H, Maurer C (2013). Subspace identification and classification of healthy human gait. PLoS ONE.

[29] Ding C, Peng H (2005). Minimum redundancy feature selection from microarray gene expression data. Journal of Bioinformatics and Computational Biology.

[30] Bus SA (2003). Ground reaction forces and kinematics in distance running in older-aged men. Medicine and Science in Sports and Exercise.

[31] Karamanidis K, Arampatzis A (2005). Mechanical and morphological properties of different muscle-tendon units in the lower extremity and running mechanics: Effect of aging and physical activity. Journal of Experimental Biology.

[32] Brach JS, McGurl D, Wert D, Vanswearingen JM, Perera S, Cham R, Studenski S (2011). Validation of a measure of smoothness of walking. Journals of Gerontology: Series A.

[33] Taunton JE, Ryan MB, Clement DB, McKenzie DC, Lloyd-Smith DR, Zumbo BD (2002). A retrospective case-control analysis of 2002 running injuries. British Journal of Sports Medicine.

[34] Srinivas M, Patnaik LM (1994). Genetic algorithms: A survey. Computer.

[35] Dorigo, M. (1992). *Optimization, Learning and Natural Algorithms*. PhD thesis, Politecnico di Milano, Italy.

[36] Kennedy, J., & Eberhart, R. (1995). Particle swarm optimization. In *Proceedings of IEEE International Conference on Neural Networks*.

[37] Geem ZW, Kim JH, Loganathan GV (2001). A new heuristic optimization algorithm: Harmony search. Simulation.

[38] Cantú-Paz E (1998). Survey of parallel genetic algorithms. Calculateurs paralleles, reseaux et systems repartis.

[39] Deng, H., & Runger, G. (2012). Feature selection via regularized trees. In *Proceedings of International Joint Conference on Neural Networks*.

[40] Eskofier BM, Kraus M, Worobets JT, Stefanyshyn DJ, Nigg BM (2012). Pattern classification of kinematic and kinetic running data to distinguish gender, shod/barefoot and injury groups with feature ranking. Computer Methods in Biomechanics and Biomedical Engineering.

[41] Chau T (2001). A review of analytical techniques for gait data. Part 1: fuzzy, statistical and fractal methods. Gait & Posture.

[42] Chau T (2001). A review of analytical techniques for gait data. Part 2: neural network and waveform methods. Gait & Posture.

[43] Sejdić E, Lowry KA, Bellanca J, Redfern MS, Brach JS (2014). A comprehensive assessment of gait accelerometry signals in time, frequency and time-frequency domains. IEEE Transactions on Neural Systems and Rehabilitation Engineering.

[44] Phinyomark A, Hu H, Phukpattaranont P, Limsakul C (2012). Application of linear discriminant analysis in dimensionality reduction for hand motion classification. Measurement Science Review.

[45] Phinyomark, A., Osis, S. T., Hettinga, B. A., & Ferber, R. (2016). Kernel principal component analysis for identification of between-group differences and changes in running gait patterns. In E. Kyriacou, S. Christofides, C. S. Pattichis (Eds.), *XIV Mediterranean Conference on Medical and Biological Engineering and Computing 2016* (pp. 586–591). Springer.

[46] Chu JU, Moon I, Mun MS (2006). A real-time EMG pattern recognition system based on linear-nonlinear feature projection for a multifunction myoelectric hand. IEEE Transactions on Biomedical Engineering.

[47] Foch E, Milner CE (2014). The influence of iliotibial band syndrome history on running biomechanics examined via principal components analysis. Journal of Biomechanics.

[48] Deluzio KJ, Astephen JL (2007). Biomechanical features of gait waveform data associated with knee osteoarthritis: An application of principal component analysis. Gait & Posture.

[49] Brandon SC, Graham RB, Almosnino S, Sadler EM, Stevenson JM, Deluzio KJ (2013). Interpreting principal components in biomechanics: Representative extremes and single component reconstruction. Journal of Electromyography and Kinesiology.

[50] Phinyomark, A., Osis, S. T., Clermont, C., & Ferber, R. (2016). Differences in running mechanics between high- and low-mileage runners. In *Proceedings of 22nd Congress of the European Society of Biomechanics*.

[51] Abdi H, Williams LJ (2010). Principal component analysis. *WIREs*. Computational Statistics.

[52] Chau T, Young S, Redekop S (2005). Managing variability in the summary and comparison of gait data. Journal of NeuroEngineering and Rehabilitation.

[53] Phinyomark A, Phukpattaranont P, Limsakul C, Phothisonothai M (2011). Electromyography (EMG) signal classification based on detrended fluctuation analysis. Fluctuation and Noise Letters.

[54] Phinyomark A, Phothisonothai M, Phukpattaranont P, Limsakul C (2011). Critical exponent analysis applied to surface electromyography (EMG) signals for gesture recognition. Metrology and Measurement Systems.

[55] Phinyomark A, Phukpattaranont P, Limsakul C (2014). Applications of variance fractal dimension: A survey. Fractals.

[56] Jitaree S, Phinyomark A, Boonyaphiphat P, Phukpattaranont P (2015). Cell type classifiers for breast cancer microscopic images based on fractal dimension texture analysis of image color layers. Scanning.

[57] Brach JS, Perera S, Studenski S, Katz M, Hall C, Verghese J (2010). Meaningful change in measures of gait variability in older adults. Gait & Posture.

[58] Chang MD, Sejdić E, Wright V, Chau T (2010). Measures of dynamic stability: Detecting differences between walking overground and on a compliant surface. Human Movement Science.

[59] Bruijin SM, van Dieën JH, Meijer OG, Beek PJ (2009). Statistical precision and sensitivity of measures of dynamic gait stability. Journal of Neuroscience Methods.

[60] Begg RK, Palaniswami M, Owen B (2005). Support vector machines for automated gait classification. IEEE Transactions on Biomedical Engineering.

[61] Kamruzzaman J, Begg RK (2006). Support vector machines and other pattern recognition approaches to the diagnosis of cerebral palsy gait. IEEE Transactions on Biomedical Engineering.

[62] Levinger P, Lai DT, Begg RK, Webster KE, Feller JA (2009). The application of support vector machines for detecting recovery from knee replacement surgery using spatio-temporal gait parameters. Gait & Posture.

[63] Kaufmann, P., Englehart, K., & Platzner, M. (2010). Fluctuating EMG signals: Investigating long-term effects of pattern matching algorithms. In *Proceedings of 32nd Annual International Conference of the IEEE Engineering in Medicine and Biology Society*, 6357–6360. 10.1109/IEMBS.2010.5627288 21096692

[64] Lee M, Roan M, Smith B, Lockhart TE (2009). Gait analysis to classify external load conditions using linear discriminant analysis. Human Movement Science.

[65] Simonsen EB, Alkjær T (2012). The variability problem of normal human walking. Medical Engineering & Physics.

[66] Mezghani N, Fuentes A, Gaudreault N, Mitiche A, Aissaoui R, Hagmeister N, De Guise JA (2013). Identification of knee frontal plane kinematic patterns in normal gait by principal component analysis. Journal of Mechanics in Medicine and Biology.

[67] Hoerzer S, von Tscharner V, Jacob C, Nigg BM (2015). Defining functional groups based on running kinematics using self-organizing maps and support vector machines. Journal of Biomechanics.

[68] Roche N, Pradon D, Cosson J, Robertson J, Marchiori C, Zory R (2014). Categorization of gait patterns in adults with cerebral palsy: A clustering approach. Gait & Posture.

[69] Kinsella S, Moran K (2008). Gait pattern categorization of stroke participants with equinus deformity of the foot. Gait & Posture.

[70] Ferrarin M, Bovi G, Rabuffetti M, Mazzoleni P, Montesano A, Pagliano E, Marchi A, Magro A, Marchesi C, Pareyson D, Moroni I (2012). Gait pattern classification in children with Charcot–Marie–Tooth disease type 1A. Gait & Posture.

[71] Giusti C, Ghrist R, Bassett DS (2016). Two’s company, three (or more) is a simplex: Algebraic-topological tools for understanding higher-order structure in neural data. Journal of Computational Neuroscience.

[72] Chan JM, Carlsson G, Rabadan R (2013). Topology of viral evolution. Proceedings of the National academy of Sciences of the United States of America.

[73] Petri G, Expert P, Turkheimer F, Carhart-Harris R, Nutt D, Hellyer PJ, Vaccarino F (2014). Homological scaffolds of brain functional networks. Journal of the Royal Society, Interface.

[74] Pokorny FT, Hawasly M, Ramamoorthy S (2015). Topological trajectory classification with filtrations of simplicial complexes and persistent homology. The International Journal of Robotics Research.

[75] Emmett KJ, Rabadan R, Ślȩzak D, Tan AH, Peters JF, Schwabe L (2014). Characterizing scales of genetic recombination and antibiotic resistance in pathogenic bacteria using topological data analysis. Brain informatics and health.

[76] Giusti C, Pastalkova E, Curto C, Itskov V (2015). Clique topology reveals intrinsic geometric structure in neural correlations. Proceedings of the National academy of Sciences of the United States of America.

[77] Petri G, Scolamiero M, Donato I, Vaccarino F (2013). Topological strata of weighted complex networks. PLoS ONE.

[78] Singh G, Mémoli F, Carlsson GE, Botsch M, Pajarola R (2007). Topological methods for the analysis of high dimensional data sets and 3D object recognition. Eurographics Symposium on Point-Based Graphics.

[79] Nicolau M, Levine AJ (2011). Topology based data analysis identifies a subgroup of breast cancers with a unique mutational profile and excellent survival. Proceedings of the National academy of Sciences of the United States of America.

[80] Lum PY, Singh G, Lehman A, Ishkanov T, Vejdemo-Johansson M, Alagappan M, Carlsson J, Carlsson G (2013). Extracting insights from the shape of complex data using topology. Scientific Reports.

[81] Carlsson G (2009). Topology and data. Bulletin of the American Mathematical Society..

[82] Ghrist R (2008). Barcodes: The persistent topology of data. Bulletin of the American Mathematical Society.

[83] Nielson JL, Paquette J, Liu AW, Guandique CF, Tovar CA, Inoue T, Irvine K-A, Gensel JC, Kloke J, Petrossian TC, Lum PK, Carlsson GE, Manley GT, Young W, Beattie MS, Bresnahan JC, Ferguson AR (2015). Topological data analysis for discovery in preclinical spinal cord injury and traumatic brain injury. Nature Communications.

[84] Guo, W., & Banerjee, A. G. (2016). Toward automated prediction of manufacturing productivity based on feature selection using topological data analysis. In *Proceedings of IEEE International Symposium on Assembly and Manufacturing*.

[85] Carstens CJ, Horadam KJ (2013). Persistent homology of collaboration networks. Mathematical Problems in Engineering.

[86] de Silva V, Ghrist R (2007). Coverage in sensor networks via persistent homology. Algebraic & Geometric Topology.

[87] Taylor D, Klimm F, Harrington HA, Kramár M, Mischaikow K, Porter MA, Mucha PJ (2015). Topological data analysis of contagion maps for examining spreading processes on networks. Nature Communications.

[88] Tausz, A., & Carlsson, G. (2011). Applications of zigzag persistence to topological data analysis. arXiv:1108.3545.

[89] Freedman, D., Chen, C., & Freedman, D. (2011). Algebraic topology for computer vision. In S. R. Yoshida (Ed.), *Computer Vision* (pp. 239–268). Nova Science.

[90] Wang J, Cazzato E, Ladewig E, Frattini V, Rosenbloom DIS, Zairis S, Abate F, Liu Z, Elliott O, Shin Y-J, Lee J-K, Lee I-H, Park W-Y, Eoli M, Blumberg AJ, Lasorella A, Nam D-H, Finocchiaro G, Iavarone A, Rabadan R (2016). Clonal evolution of glioblastoma under therapy. Nature Genetics.

[91] de Silva, V., Skraba, P., & Vejdemo-Johansson, M. (2014). Persistent cohomology for dynamical systems. In *Proceedings of 48th Spring Topology and Dynamics Conference*.

[92] Donato I, Gori M, Pettini M, Petri G, De Nigris S, Franzosi R, Vaccarino F (2016). Persistent homology analysis of phase transitions. Physical Review E.

[93] Vardaxis VG, Allard P, Lachance R, Duhaime M (1998). Classification of able-bodied gait using 3-D muscle powers. Human Movement Science.

[94] Tenenbaum JB, de Silva V, Langford JC (2000). A global geometric framework for nonlinear dimensionality reduction. Science.

[95] Bauer U, Kerber M, Reininghaus J, Bremer PT, Hotz I, Pascucci V, Peikert R (2014). Clear and compress: Computing persistent homology in chunks. Topological methods in data analysis and visualization III.

